# The Effect of a 6-Month Coach Educational Program on Strengthening Coach-Athlete Interpersonal Relationships in Individual Youth Sport

**DOI:** 10.3390/sports6030074

**Published:** 2018-07-29

**Authors:** Ausra Lisinskiene

**Affiliations:** Academy of Education, Vytautas Magnus University, Kaunas 44248, Lithuania; ausra.lisinskiene@leu.lt; Tel.: +370-650-21236

**Keywords:** coach, athlete, interpersonal relationships, intervention program

## Abstract

The purpose of this intervention study was to develop an educational program for coaches to strengthen the coach–athlete interpersonal relationship in individual youth sport. To obtain data in the qualitative interpretative phenomenology phase, 10 youth sports coaches took part in semi-structured, in-depth interviews. The educational program was developed by integrating psychological, educational and social skills into the educational coaching sessions. The program involved a detailed video analysis, theoretical classes, and individual consultations. The qualitative interpretative phenomenology research design was used and enabled to evaluate the program. The study results revealed that the program had a positive impact on the transformation of the coach–athlete interpersonal relationship in sport. Behavioural, emotional, cognitive, and social strategies changes occured. The quality of the coach–athlete relationship changed: trust, communication, cooperation, encouragement, and a connection between athletes and the coaches appeared. The study’s results showed that the educational program for coaches had a positive effect on the quality of interpersonal relationships between athletes and the coaches and increased positive coaching strategies in youth sport.

## 1. Introduction

Coaches are playing an increasingly important and diverse role in sport [1]. The coach is one of the main figures in sports that has a direct influence on athletes’ participation [2]. Coaches are not only expected to coach technically and tactically but also to coach and help a person to develop physically, emotionally, socially, and cognitively [1]. Coaches create a suitable environment that promotes the development of athletes’ life skills [3]. The relationship between the coach and the athlete has been recognized as an important factor in creating positive educational sports experience for athletes [1,2,3,4,5,6]. Coaches’ behaviours, attitudes, and values are imitated by their players not only in sports settings but in other contexts of players’ lives too [2]. However, recent studies show that most children receive coaching from untrained amateur coaches [4]. Educational coaching strategies can be more or less effective. Scientific research results [7,8,9] show that most athletes prefer an environment that promotes education in both sports participation and personality development. Coaches who are supportive, knowledgeable about the sport they are coaching, that prioritize athlete development, and are good motivators receive greater recognition. Conversely, coaches who offer little or no support, prioritize winning, are not organized, and have limited knowledge of the sport they are coaching are seen as ineffective. In this sense, the interpersonal relationship between the coach and the athlete is of highest importance, because it can influence an athlete’s motivation in sport. According to Duda’s conceptualization, the coach-created motivational climate should be considered as multidimensional and can be empowering or disempowering [9,10]. An empowering coach-created motivational climate is characterized by lower-order, task-involving, autonomy-supportive, and socially supportive features. In contrast, a disempowering climate is marked by lower-order, ego-involving, and controlling (including those which are relatedness thwarting) characteristics.

Despite the fact that coaches play the most important role in motivating youth, athlete motivation to participate in sport largely depends on his/her personal motivation. Scientific research has shown that some athletes are engaged in sport by external factors, such as pressure from their coach, parents, reward systems, and recognition. Other athletes are involved by curiosity and interest, a desire for growth and improvement, and a desire to master new skills [11]. In order to better understand the coach–athlete relationship and the athlete decisions in sport, this study draws upon self-determination theory. Self-determination theory (SDT) is a theory of human motivation and personality that concerns people’s inherent growth tendencies and innate psychological needs. As the athlete’s motivation is considered to be the most important factor for participation in youth sport, the reasons why some athletes demonstrate a passionate desire to improve their sport skills and others do not are unclear. SDT focuses on the degree to which an individual’s behaviour is self-motivated and self-determined. According to SDT, the three basic psychological needs, which are said to be innate, universal across cultures, and evident in all developmental periods, can aid in this internalization process. The three needs are competence (when an individual has the opportunity to seek challenges and demonstrate their capacities), relatedness (when an individual experiences a sense of belonging with others), and autonomy (when an individual acts in line with his or her own interests and values) [12]. Understanding why the athlete is engaged in sport activities and which factors are determining the choice between individual or team sport are complex phenomena. The coach as an educator must find the answer to such related questions. This is why coach–athlete interpersonal relationship research is one of the central areas in social sports psychology [8,9,10].

The coach–athlete relationship that contains elements of success and effectiveness is the ideal athletic relationship. The coach–athlete interpersonal relationship includes performance success, as reflected in improving a skill or achieving success, and personal growth, as reflected in experiencing a sense of maturity and satisfaction [9]. Most research studies outline that the characteristics of an expert vary between the skills, knowledge, and experiences, namely, competencies. One of the most important sources of learning for the coach is the workplace. Within the coaching environment, coaches have opportunities to reflect on their practice, discuss issues with their athletes, observe other coaches, and draw upon their previous coaching or playing experiences [13]. Research shows that successful coaches have attended more coach education programs and courses, spent more time learning from others, and encountered and solved more problems, and, as a result, have had many opportunities to enhance and deepen what they know about coaching, including the tactical components of coaching [14]. It appears that expert coaches have more coaching knowledge than novice coaches and that, as coaches become more expert, the role of tactical knowledge plays a greater role in their coaching practice. Understanding how the coach’s knowledge is both developed and applied is a key consideration for those responsible for developing coach education programs. Researchers [2,13,15] highlight the need to develop training programs for coaches in order to promote the involvement of players. Coach education programs tend to focus on coaching theory, sport-specific techniques, tactics, and supervised practice. However, they are often criticized for their inability to modify coaches’ behaviour or philosophies once they have returned to their real coaching situation. More cognitively challenging coach education programs appear to carry greater potential for providing coaches with knowledge, including tactical knowledge, and skills to transform their coaching practice [10]. However, psychological, educational and social skills that would be integrated into the coaching environment are lacking. Psycho-behavioural development [15] can be taken to reflect the operationalization of psychological skills (such as goal setting, focus, and distraction control) to self-regulate observable learning and performance behaviours. As such, it relates to people exhibiting psychological skills through behavioural outcomes.

Given that there is a need for applied research to create a better understanding of (1) how the long-term educational program developed for coaches could influence the change in coach–athlete relationship and (2) how the integration of psychological and educational skills into the educational program effects coaching experiences in youth sport. Therefore, the purpose of this six-month intervention study was to develop an educational program for coaches. The second aim was to evaluate the change in the coach–athlete interpersonal relationship in sport based on a 6-month educational program. This study is based on the integration of psychological, educational and social skills into a coaching program and aimed at presenting a 6-month coach intervention program while examining the changes in the coach–athlete relationship in sport.

## 2. Materials and Methods

*Qualitative research design.* Interpretative Phenomenological Analysis method, hereinafter IPA [16,17,18], was selected from all available qualitative research methods. IPA has a double purpose: It is both data collection and qualitative data analysis method [18]. Interpretative phenomenological analysis [17] focuses on the lived experience of participants by incorporating phenomenology and interpretation. It shares the aims of idiographic phenomenology, which provides a detailed analysis of elements of the reflective personal and subjective view of individual experiences. IPA moves one step beyond phenomenology (participant’s accounts) and attempts to report on the participant’s experience by considering the researcher’s view of the world during interpretation.

*Selection of research participants.* The research participants were chosen from volunteers who answered the invitation to participate: Coaches of an individual sport. Demographic characteristics included a number of research participants, coaches’ gender, age, and education level. Moreover, the demographic data included a sport type. Finally, sport coaching experience in years was included. In the survey that lasted for almost two years, ten stories of coaches were voice recorded. The sample consisted of 10 coaches who were 42 years old in average. Three females and seven males were research participants in this study. All of the coaches were highly educated and were of a higher master degree education level. All 10 coaches had an average of 12 year coaching experience in individual sports of martial arts. The coaches involved in this study were focused on coaching adolescent athletes aged 12–17. Each of the coaches had at least three athletic groups in each age group: 12–14 age of group, 15–16 age of group, and 16–17 age of group. Each group consisted of at least of 10 adolescent athletes. In total, each coach was coaching 90 adolescent athletes in average.

### 2.1. The Process of the Research

*Research procedure.* Regarding the sensitive topic of analysing coach–athlete interpersonal relationships in youth sports, the researcher carefully planned the research process in the following stages. First, researcher obtained ethical approval from Vytautas Magnus University about the eligibility to conduct the research. Next, the researcher organised a meeting with coaches and provided a detailed explanation about the ongoing research and the program. Finally, coaches’ consent to participate in the study was obtained prior to the planned research. Below, all steps are explained in more detail. With the help of administration of sports clubs, information about the planned survey was announced in seven private sports clubs of martial arts in Kaunas, Lithuania. Each private sport club had 6 coaches on average. Only the coaches interested in the survey collected flyers with the description of the study and researcher’s contact details. The coaches were given all the researcher’s contacts, enabling them to ask questions at any time. Thirteen coaches expressed the wish to participate. However, based on research participant selection criteria—homogeneity, information coverage, and informed consent to participate in the survey—three participants were excluded from the research. The information about the ongoing research and developed educational program was clearly explained to all coaches in the meeting at arranged time. The author of this program, with the help of sports club administration, has organized the first visit. All ten coaches were invited to the meeting. The author of the program described the program in a very careful manner. Research participants were informed about the ongoing research and developed educational program. Research participants were informed about the aim of the program, the content, and the structure of the program. Coaches were informed about the benefits they might gain after the program. Research participants were also informed about the responsibility to take part in the research and the program. All ten respondents were invited for an interview prior the intervention and post-intervention. Questions given to the respondents were formulated in accordance with the research problem. The plan of questions was followed in order not to digress from the research issue and at the same time not to restrict free associations of the respondents and the content of their stories.

*Interview Protocol Development.* The questions given to the research participants focused on the issues raised by the investigation. The protocol served only as a guide in each interview to prevent the researcher from uncontrolled deviation from the analysed topic, and to restrict free associations of the participants and the content of the narrative. Semi-structured interview questions began after the researcher established consent. Next, the participants shared more about themselves, their families, and hobbies. Later, more sensitive questions related to the research emerged, such as “How do you understand the coach-athlete relationship?” or “What difficulties in coach-athlete relationship have you faced during your coaching experience?” The interviews concluded with a neutralizing inquiry about their feelings after the meeting, and an opportunity to ask questions to the researcher.

*Semi-structured interviews.* Information about coach–athlete interpersonal relationships in sport was collected by means of semi-structured interview. This approach is considered to be an appropriate data collection method in which the researcher attempts to get to the depth of meanings and the survey is first of all focused on insights and understanding. Interviews were conducted in the author’s research office, and the schedule was organized in separate individual meetings with coaches, scheduled in advance at a time convenient for all parties. Interviews lasted from 1 h to 1.20 h.

*Research data analysis.* Data analysis was carried out in compliance with the methodological requirements of interpretative phenomenological analysis [18]. The analysis contained the following stages: transcription, analysis, and credibility checks. Each interview was audiotaped and transcribed verbatim [18]. At this stage, the focus was on how the participants talked about themselves: their tone, rhythm, pauses, or changes in topics. IPA requires detailed and comprehensive interview transcription material (text), which is the object of the analysis. Therefore, some essential aspects of participant interactions were noted (laughing, crying, silence, change in mood, etc.). The Material collected in 10 interviews—voice records (more than 10 h) were transcribed into text. While analysing the results, the coding system of the qualitative study included the changed research participant name. The name of the research participant was changed and identified, for example, “Coach 1” (where, 1—the number of interview). Author followed the ethical requirements for qualitative research [18] in order to ensure and maximize the security of research participants’ identification.

*Ethical aspects of the survey.* The Survey respondents participated voluntarily and for no remuneration. They did not receive any misleading information regarding research goals or the form of results presentation. The research was conducted by the following principles [19]: Right for protection from damage, right for safety, usefulness of the survey, privacy, confidentiality, and fairness.

The ethical principles included obtaining individuals’ consent. The consent included information about the ongoing research. The aim and the purpose of the study were explained. The use of research participant-given information for research purposes was explained. The right to refuse to participate in the research at any time was explained as well. In this sense, the research participant would be excluded from the research and the recorded interview automatically deleted.

*Research quality assurance.* Researcher followed [20] four principles of research quality assessment: (a) sensitivity to context, (b) commitment and rigour, (c) transparency and coherence, and (d) impact and importance. Applying sensitivity to context, researcher treated her research participants as true research experts, without researcher’s power-play interruptions. The interpretation of the results was made by giving participants a voice in the project and allowing the reader to check the interpretations made. Regarding commitment and rigour, researcher showed a high degree of attentiveness to the participant during data collection and care with which the analysis of each case is carried out. To conduct an in-depth IPA interview requires a considerable personal commitment and investment by the researcher to ensure the participant is comfortable, and to attend closely to what the participant is saying. Rigour refers to the thoroughness of the study, for example, the quality of the interview and the completeness of the analysis undertaken. The quality of the interviews was planned carefully. The methodological consultations, as well as seminars and workshops, supervisions, and trainings with experts in IPA, were attended prior to the research. Researcher carefully reviewed every interview and critically reflected on each. Applying the principle of transparency and coherence, researcher clearly and carefully described the research process, including how participants were selected, how the interview schedule was constructed, how the interviews were conducted, and what steps were used in analysis. The coherence is seen through themes brought together logically through drafting and redrafting. Finally, impact and importance are broad principles seen through real validity, which lies in whether the findings tell the reader something interesting, important, or useful [20]. Researcher presented a true story of coaches’ experiences in relation to their interpersonal relationships to athletes. This study tells the reader about the possible interventions through coaching experiences in youth sport.

### 2.2. Intervention Program

**Figure 1:**
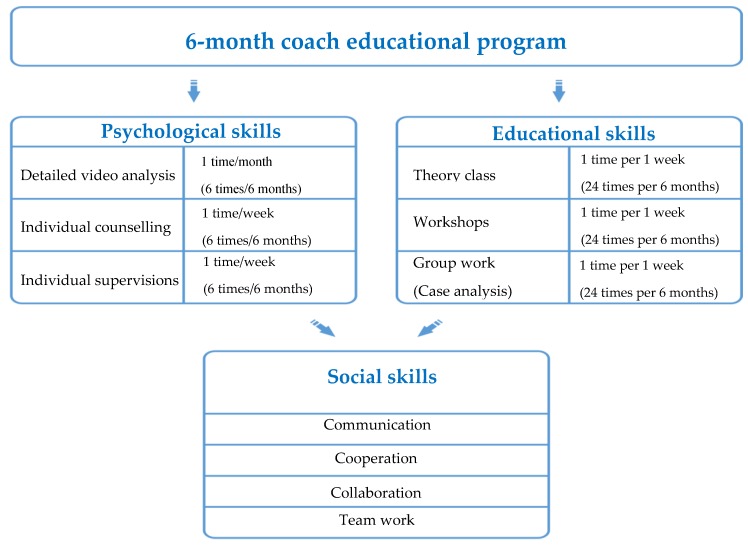
The description of 6-month coach educational program.

A 6-month coach education program (Figure 1) was developed based on qualitative research results (prior intervention) (Table 1). The program was implemented by choosing one Lithuanian private sports club. The invitation for the coaches to participate in the program was conducted. Coach education program was developed by integrating psychological, educational, and social skills into general sports club programs. The program was based on practical and theoretical classes attended by coaches. Sports psychologist and sports educator was involved in the program. Coaches participated in a program once in a week per 6-month period.

*Psychological aspect.* An important psychological element of the program included a detailed video analysis of the training class. The aim of this video analysis was to examine the videotaped process of a sport training session, to observe coach–athlete relationship in training sessions. The instructions on how to be prepared to participate in video analysis in the training session were given. All the athletes who participated in this analysis and the athletes’ parents signed an agreement expressing their consent to participate in such analysis. Through detailed observation, the emotions and behaviour of coaches and athletes at training classes were discussed in a workshop session. The videotaped class was also discussed in separate individual meetings with coaches scheduled in advance. The psychological aspect was ensured by involving individual counselling and personal supervisions. A preferred counselling schedule has been identified. Individual coach counselling and personal supervisions included individualized coach experience analysis, and/or specific interpersonal relationship case analysis.

*Educational aspect.* The program involved educational aspect, a theory class led by sports psychologist and sports educator for coaches (once in a month/one academic hour) to help them understand the benefit of the educational coaching process. The following themes were covered in the coach training session: The importance of the coach–athlete attachment in youth sport, the importance of coach’s psychological knowledge in youth sport, the importance of coach’s educational knowledge in youth sport, the importance of coach’s social knowledge in youth sport, involved/overinvolved/not involved coach, and positive/negative coaching strategies in youth sport. The topics were formulated and emerged from the background of systematic scientific literature review. They also emerged from the author’s research background. Finally, the topics emerged from the author’s athletic and coaching practical experience background. The educational aspect of the educational program used for the intervention also involved workshops for coaches, and psychological, educational, and social skills were trained during workshops. The workshops included different situation analysis, video analysis, and group work. A schematic educational program description is presented in Figure 1.

Figure 1 illustrates six months duration of educational program for coaches. The figure illustrates how psychological, educational, and social skills were integrated into the education program. The education of psychological skills included a detailed video analysis of the coaching process. In total, each coach, out of 10, represented 6 training sessions that were video recorded. Video analysis was carried out 1 time per month. Later on, a video recorded analysis was discussed and analyzed during individual supervisions (1 time per week), and in workshops (1 time per week). Individual counselling was planned in advance by representing a schedule time.

Educational skills were trained through the context of theory class, which included 1 topic per month. However, this topic was analyzed in 4 weeks. In total, 6 topics were analyzed in depth. As was mentioned earlier, educational workshops included a detailed video analysis of each case. The educational aspect included group work that was organized one time per week. The group work included a specific case analysis that was based on real coaching practice. For example, each coach represented a specific case or situation in relation to coach–athlete relationship issues. The group analyzed the possible best solution of particular case. In total, 90 h were included in the educational program. In addition, social skills were integrated in psychological and educational aspect. In long term, coach training development communication, cooperation, collaboration, and team work occurred.

## 3. Results

Results of designed educational program are presented in Table 1 and Table 2. Prior to the intervention, researcher conducted interviews in order to find out what specific skills and knowledge coaches needed in order to enhance the educational interaction and interpersonal relationships between the coach and the athlete. Table 1 presents the results of the qualitative study and the coaches’ responses to the specific knowledge they need. Based on the study results presented in Table 1, the researcher designed the educational program for coaches. After the 6 months of the intervention period, as a follow up, the researcher conducted interviews with the participants in order to find out the effectiveness of the program. The coaches, i.e., the research participants, were treated as true experts in the evaluation of the program. The evaluation of the program, and the themes that emerged from the qualitative research after the program, are presented in Table 2.

### 3.1. The Change of Psychological Skills

Conversations with coaches revealed that psychological skills were the most important parts of the sessions in the entire six-month program. Prior to the intervention, the coaches expressed their feeling that they lacked psychological skills in their coaching experience. To strengthen athlete motivation was very important to them. The coaches stated that athletes’ motivation to participate in sports is the most important aspect of sports. Some athletes have intrinsic motivation and others do not. Themes such as the ability to inspire the athlete, amotivation, and demotivation appeared in their relation to athlete context. Athlete goal setting and self-determination as important element in sport also were difficult to achieve. Emotional skills such as fair play, and stress management prior, during, and after competitions were difficult to control. As a result, coach–athlete behaviour strategies were weak, which caused such feelings as alienation, disinterest, and relationship distance between the coach and the athlete. Such knowledge is important in strengthening coach-athlete relationship. Higher education helped them to gain generic skills useful in everyday coaching practice; however, the psychological skills need to be developed further:

“It was a very deep look into the coaching. As for me, I always missed this part of my coaching experience. The important moment is that coach-athlete relationship depends on the coach’s attitude towards the athlete, the ability to say tactfully, in a very common sense what you want to say to an athlete, and not too much. Because the “word” could be much stronger than a physical moment.”

“I understood now, that the psychological, educational training sessions for coaches need to be continually organized. This six-month program is a first step into the coaches’ professional development. Sports psychologist, sports educator and sports coach should collaborate continuously to achieve the best results.”

“Technical, tactical skills are very important. But the psychological moment is the most important, and everything actually starts from this point. If you have a good, secure relationship with your athlete, you know, as a sport professional the technical, tactical part will be successful. That‘s what I realized.”

“A coach needs to know a lot. He is a sports psychologist, sports educator, sports coach as a professional; he is a mother, a father, a friend, a colleague, he is everything to the athlete. But what I learned from this program is that to maintain all these relationships you as a coach need to work hard to be the authority to them.”

Coaches with a great coaching experience honestly expressed their warm feelings, and that communicating with an athlete is not the same as communicating with a friend or a family member. They must inspire, teach, educate, and communicate. They mentioned that coaching is not a job. It is life. The learning process never stops. The need for developing psychological skills was emphasised. How to act in a certain situation is hard for them choose. Every athlete is a personality. There is a need to look at every athlete as a separate personality. This task requires deep psychological knowledge. Every athlete for a coach means, firstly, raising a thoughtful personality for life, and only the second goal is to inspire sport values, sport education, and technical and tactical sport aspects. They mentioned that a coach for an athlete means a lot. In the long-term, a coach in youth sport becomes like a second parent, an important family member, a positive role model, and a positive authority. In this sense, a coach must be a positive role model for every athlete.

### 3.2. The Change of Educational Skills

The research showed that the expression of the secure coach–athlete relationship depends on how the coach perceives the value of sport and how they transfer this perception into the athlete’s sporting life. The interviewed coaches acknowledged gratefully that the educational part, meetings with athletes, and discussing different topics with their athletes is necessary. However, all coaches emphasized that adding educational sessions to their coaching experience is challenging:

“Honestly, I never did any educational meetings with athletes before. The time issue. We, of course, meet and discuss pre-competition session, post-competition session. But such meetings, as you know, have the goal to educate athletes, for example, what is the sporting phenomenon, how not to burn out in sport, what are the drop-out prevention strategies in sport, what is the emotion managing in sport, —we never discussed.”

*(Coach 3)*

“I now see how important it is. I actually organized the meeting with athletes as the program suggested. I gave a lecturer, held a seminar for them. They were really interested. Actually, what I felt is that I know so little. I had to prepare, to read information about a particular topic that I want to talk with them, I had to manage time, reflection, discussion. I realized how much I as a coach need to learn first.”

Professionalism in every context is highly appreciated. The coaching context is no exception. To be a good professional in a field means to gain deep knowledge, to keep on learning, especially in the education context, in which you have to be prepared to act in a way that this acceptable to each generation. Meetings not only with athletes but also with parents have created a motivational, friendly environment, based on trust and reliability. However, coaches mentioned that organizing educational meetings or theoretical seminars by presenting and educating athletes on how to improve technical and tactical skills were a challenge to them.

### 3.3. The Change of Social Skills

Prior intervention research results showed (Table 1) that coaches felt the lack of social skills learning strategies. Reflecting on coaches’ thoughts, and by interpreting research results, it is important to mention that social skills are particularly important for those specialists who intensively and directly work with people. The ability to communicate, to receive and to give feedback, to reflect, and to interact are key components of a coach. To instigate and inspire good social habits, such as social values, and sport values to every athlete is a challenging task as well. It seems that coaching is one of those contexts where coach’s work is similar to that of a teacher. The responsibility to raise the athlete as a personality first and only then as an athlete is an important factor in their work experience. To explain the importance of responsibility; fair play; and respect to the opponent and the teammates, or coaches, parents, and other important members, ethical aspects of sport are some of the most important aspects in sports environment. For coaches, especially for those who work with adolescence age athletes might be a challenging issue. At this stage, athletes are going through rapid biological, cognitive, and physiological changes associated with puberty, which might affect the motivation to participate in sporting activities and also might affect the sporting results. Coaches with poor social and emotional skills face greater risks in communication, interpersonal relationships, cooperation, and collaboration managing, which in turn can lead to negative youth experience in sport, dropping out, or burnout. In addition to difficulties experienced in communicating with young athletes, coaches acknowledged that communicating with other sport participants is also challenging. Working with athletes’ parents, other coaches, sport administrations, referees, etc., takes time and requires knowledge and diplomacy:

“The big issue is parents. I want to do my best with athletes, but what I realised that if I want to do my best with athletes, I need to have best relationship with athletes’ parents as well. To communicate with parents is challenging.”

“After the educational classes, analysing different situations of positive and negative examples of all three members of the sport, namely coach-athlete-parent, made sense. I now know that responsibility lies upon all three members, not only the coach. That is surprising. Also, what I think, the intervention is strongly needed for the parents’ involvement into youth sport.”

“After the program and the sessions on social skills development I learned that the communication and relationship between all the members directly influences the young athlete. It is unbelievable. But it’s true. I thought that close relationship between the coach and the athlete is the most important. But actually you need to see who is around you as well.”

Coaching is one of those educational contexts in which a coach needs to have educational interaction skills not only to deal with the athlete but also with other important members, such as athlete’s parents, other coaches, and sports organization. Communication skills need to be developed. Research participants reflected that interaction skills, especially with athletes’ parents, has increased. To maintain a positive relationship with athletes’ parents is a challenging issue, mentioned the coaches. After the educational program, the sport environment and relations with other sport participants became more flexible, positive, and friendly.

## 4. Discussion

The purpose of this intervention study was to develop an educational program for coaches with the aim to strengthen coach–athlete interpersonal relationship in individual youth sport. The program was developed based on the evidence of scientific research, the program author’s scientific knowledge and her past research, and on her long-term sport and coaching experience. In order to improve the aforementioned aspects, before the intervention the researcher conducted the first qualitative phenomenological analysis. For the researcher, it was important not to bring her own sport and coaching experience while conducting research so that it would not influence the study results. The researcher was highly psychologically prepared without holding any preconceived assumptions regarding its truth or usefulness.

The researcher conducted in-depth interviews with coaches in order to find out the most important issues for coaches and the difficulties faced in coach–athlete interpersonal relationship. Prior to the intervention, the following major themes emerged: “the need for psychological skills” with sub-themes such as “strengthening athlete motivation”, “strengthening athlete emotional skills”, and “srengthening coach-athlete behavior strategies”; the second major theme “the need of educational skills” with sub-themes “strengthening interaction skills” and “strengthening interaction skills”; and the third theme “the need of social skills” with sub-themes “strengthening social sensitivity feeling” and “implementation of social values”. Based on these results, the intervention program was developed by the author, who is both a practitioner and a theoretician in the field of sports education and sports psychology.

The first theme “psychological skills” described how coach–athlete interpersonal relationship occurred. Coach–athlete motivation increased, and coach–athlete emotional skills in a sports environment, such as emotions pre-competition, during competition, and post-competition, became more stable. Emotion control mechanisms developed. Coach–athlete behaviour strategies, such as harmonious coach–athlete relationship, understanding, trust, and feeling of closeness appeared. Finally, individual supervisions and conversations with sports psychologist enabled to provide support for the coach in understanding the overall coach–athlete relationship. The important finding of this study, namely, the psychological aspect in youth sport, addresses the need for strong psychological skills in the coaching context. This study supports other research findings that the psychological skills are of the highest importance [21]. The psychological climate created in the sport environment based on these study results is of high relevance. Other scientific studies have also demonstrated that athletes who possessed psychological coping skills exhibited greater athletic success [22]. Authors [23] examined whether coaches employ psychological skills, and where, when, and for what purposes they use them. The coaches reported a more frequent use and for greater number of purposes for using self-talk and imagery than relaxation and goal setting. Researchers emphasized the findings and suggested that coaches should employ psychological skills; it is imperative that they become aware of what skills they require and what skills they possess if they are to maximize their use across their wide-ranging coaching roles. The study of [2] made an evaluation of the verbalizations of the trainer in a competition, along with a behavioural program. The authors observed the behavioural changes in the trainer and examined if these changes influenced the satisfaction of footballers. The results showed a decrease in coaches’ negative verbal behaviour and an increase in the athletes’ satisfaction after the behaviour training program, no matter the level of the competition.

The second theme, “educational skills”, describes how coach–athlete interpersonal relationships strengthen after the intervention. Six months is quite a short period to perceive noticeable results. The progress of educational skills improvement was observed. The interaction between the coach and the athlete emerged. “Cautious” interpersonal relationships between the coach and the athlete occurred. The term “cautious”, as the coaches described it, means very thoughtful access to the athlete. They mentioned that after the intervention, they became more diplomatic and tolerant towards athletes and used more tactful access to the athlete. In this sense, self-determination theory helps to explain the results of how coach–athlete relationship occurs and athletes’ decisions in sport. Three basic psychological needs, competence (when an individual has the opportunity to seek challenges and demonstrate their capacities), relatedness (when an individual experiences a sense of belonging with others), and autonomy (when an individual acts in line with his or her own interests and values) have been strengthened. According to interviewed coaches, theory-based skills were improved. Especially, links between theory and practice occurred. The workshops enabled one to analyse real situations that occur in the sporting environment.

Continuing discussion, [23] presented high-performance coach education program, hereinafter CEP. The findings from this study suggest that by conceptualizing the coaching process “coaching as education”, the participants began to expand their coaching knowledge base and incorporate wider educational views of the coaching process. In particular, coaches began to deconstruct their previous experiences of CEP and validate a need to move beyond the technical skills development model. Of particular value to the participants in this study was an understanding of educational learning theory and appropriate coaching applications, athlete empowerment models, and the value of reflective practice as a means of forming an ever-evolving coaching philosophy.

The third theme, “social skills”, describes how coaches improved their skills in the social context. The intervention program integrated psychological and educational skills to improve social skills learning. Social sensitivity emerged after the intervention. Coaches acknowledged the importance of social interaction, which they perceived as social sensitivity, enabling them to develop positive relationship between the members of the sporting activity. Social sensitivity helped to develop social values such as fair play, respect, trust of people, responsibility, and thinking about career opportunities, which, as coaches admitted, still need to be deepened. The research results [13] show that sports coaches aim to create meaningful sporting experiences for youths. These meaningful sporting experiences were considered a precondition for keeping youths engaged in the sporting activities, as well as a precondition for life skills development. The sports coaches specifically focused on creating little moments of success and on making sure that young athletes felt they belonged to a group. To ensure that the young people could experience moments of success, specific coaching strategies were implemented to increase the adolescents’ comprehensibility and manageability in specific sports situations. According to sports coaches, experiencing little moments of success could contribute to an increase in socially vulnerable youths’ understanding of everyday challenges that they face, as well as contribute to their ability to deal with these challenges. Creating meaningful sporting experiences may help youths “to learn to cope”—a skill that could be beneficial over their lifespan and in different societal domains. The author of [24] showed that coach, as well as youth sport parent educational programs, are needed to enhance and to maximize youth sport participation. Stable coach–athlete interpersonal relationship and the professionalism of the professionals makes a sport club reliable. In relation to this, other researchers have found that the context of perceived quality and user satisfaction of sports facilities ensures results in the image of the organization as well [25,26]. Another case study by the authors of [26], assessing the satisfaction of the users of facilities, showed the highest values of satisfaction, which were given for relationship between the staff. This means that relationship between sport club members and the quality of social interaction are important factors that influence the overall coach–athlete relationship.

The present study has presented a program on an educational level. The program improved a positive effect on coach education and enabled one to integrate psychological/educational/social skills and make a positive impact on the interpersonal relationship between the coach and the athlete. However, it is important to mention that this program was designed for a 6-month educational period. Therefore, as the results show, there is a need to continue the educational program on a longitudinal level (a period of one or two years or more) in order to evaluate the coach–athlete interpersonal relationships and overall effectiveness of the program. In addition, this intervention program included 10 youth sport coaches. The use of 10 coaches is a limitation, because the sample size is small with regard to the evaluation educational program; even the design is qualitative.

## 5. Conclusions

The study results revealed that the educational program developed for coaches had a positive impact on the evolution of the coach–athlete interpersonal relationship in sport. The changes in psychological, educational, and social strategies used by coaches were achieved. Psychological aspects such as coach–athlete emotional intelligence, motivation, and behaviour strategies have improved. Educational skills improved through educational interaction with athletes and their parents. Theoretical knowledge has been deepened, resulting in young athletes’ development as well. Improved psychological and educational skills have made a positive impact on social skills learning. The development of social sensitivity and social values have been identified. The study results revealed that the educational program for coaches had a positive effect on the quality of interpersonal relationships between the athlete and the coach and increased positive coaching strategies in youth sport.

## Figures and Tables

**Table 1:** Prior intervention: Theme table.

Major Themes	Themes	Subthemes
**Psychological skills needed** **Lack of psychological skills**	Strengthening athlete motivation	Ability to inspire the athlete
		Amotivation/demotivation
		Goal setting
		Self-determination
	Strengthening athlete emotional skills	Ability to regulate emotions
		Aggression
		Fair
		Stress
	Coach-Athlete behaviour strategies	Feeling of alienation
		Ignoring
		Relationship distance
**Educational skills needed**	Strengthening interaction skills	Verbal communication
		Educational methods
		Educational strategies on communication
	Strengthening theoretical coach background	Difficulty to put theory into practice
**Lack of social skills**	Strengthening social sensitivity feeling	Ability to interact with parents
		Ability to interact with other coaches
		Ability to interact with sports
		federation administration
	Implementing social values	Fair play
		Respect
		Communication
		Responsibility

**Table 2:** Post-intervention: theme table.

Major Themes	Themes	Subthemes
**Psychological skills**	Coach-Athlete motivation	Goal setting
		Self-determination
		Motivation to gain recognition
		Engagement
		Enjoyment
	Coach-Athlete emotional skills	Anger management
		Fair management
		Stress management
		Conflict management
	Coach-Athlete behaviour strategies	Harmonious coach–athlete relationship
		Understanding
		Trust
		Communication
		Closeness
		Concern
		Connection
		Cooperation
**Educational skills**	Interaction skills	Coach–athlete educational interaction
		Coach–parent educational interaction
		Coach–athlete–parent interaction
	Interpersonal skills	Communication skills
		Variety of educational methods appeared
		Variety of educational strategies on communication skills appeared
	Theory-based skills	Efforts to link the theory with practice
		Strengthened theoretical background

## References

[1] Christensen M. (2014). Exploring biographical learning in elite soccer coaching. Sport Educ. Soc..

[2] Ortín F.J., Maestre M., García-de-Alcaraz A. (2016). Football coaches training and satisfaction of young athletes. SPORT TK Revista EuroAmericana de Ciencias del Deporte.

[3] Camiré M., Trudel P., Forneris T. (2012). Coaching and Transferring Life Skills: Philosophies and Strategies Used by Model High School Coaches. Sports Psychol..

[4] O’Connor D. (2011). Enhancing coach-parent relationships in youth sports: Increasing harmony and minimizing hassle. Int. J. Sports Sci. Coach..

[5] Nash C. (2015). Practical Sports Coaching.

[6] Smoll F.L., Cumming S.P., Smith R.E. (2011). Enhancing coach-parent relationships in youth sports: Increasing harmony and minimizing hassle. Int. J. Sports Sci. Coach..

[7] Camiré M., Forneris T., Trudel P., Bernard D. (2011). Strategies for Helping Coaches Facilitate Positive Youth Development through Sport. J. Sports Psychol. Action.

[8] Jowett S., Poczwardowski A. (2007). Understanding the Coach-Athlete Relationship. Social Psychology in Sport.

[9] Duda J.L., Balaguer I. (2007). The coach created motivational climate. Social Psychology in Sport.

[10] Appleton P.R., Ntoumanis N., Quested E., Viladrich C., Duda J.I. (2016). Initial validation of the coach-created Empowering and Disempowering Motivational Climate Questionnaire (EDMCQ-C). Psychol. Sport Exerc..

[11] Vallerand R.J., Salvy S.J., Mageau G.A., Elliot E.J., Denis Frédéric P.L., Grouzet M.E., Blanchard C. (2007). On the Role of Passion in Performance. J. Personal..

[12] Ryan R.M., Deci E.L. (2000). Self-determination theory and the facilitation of intrinsic motivation, social development, and well-being. Am. Psychol..

[13] Hall E., Gray S., Sproule J. (2015). The microstructure of coaching practice: Behaviors and activities of an elite rugby union head coach during preparation and competition. J. Sports Sci..

[14] Lisinskiene A. (2016). The educational interaction between adolescents and parents in sporting activities. A Dissertation.

[15] MacNamara Á., Collins D. (2010). The role of psychological characteristics in managing the transition to university. Psychol. Sport Exerc..

[16] Smith J.A. (1996). Beyond the divide between cognition and discourse: Using interpretative phenomenological analysis in health psychology. Psychol. Health.

[17] Smith J.A. (2011). Evaluating the contribution of interpretative phenomenological analysis: A reply to the commentaries and further development of criteria. Health Psychol. Rev..

[18] Smith J.A., Flowers P., Larkin M. (2009). Interpretative Phenomenological Analysis: Theory, Method, and Research.

[19] Sacks D., Westwood M. (2003). An approach to interviewing adolescents. Paediatr. Child Health.

[20] Yardley L. (2000). Dilemmas in qualitative health research. Psychology and Health..

[21] Du Plessis Y., Barkhuizen N. (2012). Psychological capital, a requisite for organizational performance in South Africa. S. Afr. J. Econ. Manag. Sci..

[22] Thelwell R., Lane A., Weston N., Greenlees I. (2008). Examining relationships between emotional intelligence and coaching efficacy. Int. J. Sport Exerc. Psychol..

[23] Galvan H., Fyall G., Culpan I. (2012). High-performance cricket coaches’ perceptions of an educationally informed coach education program. Asia-Pac. J. Health Sport Phys. Educ..

[24] García Mayor J., Vegara Ferri J.M., López Sánchez G.F., Díaz Suárez A. (2016). Satisfaction of sports services users in Orihuela (Alicante). SPORT TK Revista EuroAmericana de Ciencias del Deporte.

[25] Aparicio Sarmiento A., Gil López M.I., López Sánchez G.F., Díaz Suárez A. (2016). Satisfaction of users of two padel clubs in Cartagena (Region of Murcia). SPORT TK Revista EuroAmericana de Ciencias del Deporte.

[26] Sánchez García C., González Carcelén C.M., López Sánchez G.F., Díaz Suárez A. (2017). Satisfaction of external customers. A case study of an indoor swimming pool. SPORT TK Revista EuroAmericana de Ciencias del Deporte.

